# Aqueous extract of *Peperomia pellucida* (L.) HBK accelerates fracture healing in Wistar rats

**DOI:** 10.1186/s12906-017-1686-3

**Published:** 2017-04-04

**Authors:** Ngueguim Tsofack Florence, Sakouong Talle Suewellyne Huguette, Donfack Jean Hubert, Gounoue Kamkumo Raceline, Dzeufiet Djomeni Paul Desire, Kamtchouing Pierre, Dimo Theophile

**Affiliations:** 1 Department of Animal Biology and Physiology, Faculty of Science, University of Yaounde1, P.O. Box 812, Yaounde, Cameroon; 2 Department of Biomedical Sciences, Faculty of Science, University of Dschang, P.O. Box 67, Dschang, Cameroon

**Keywords:** Drill-hole, *Peperomia pellucida*, Alkaline phosphate, Fracture healing, Haematological parameters

## Abstract

**Background:**

*Peperomia pellucida* (L.) HBK is consumed as vegetable and used in Cameroonian traditional medicine for the management of diseases and for fracture healing. Therefore the aim of this study was to evaluate the effects of the aqueous whole plant extract of *Peperomia pellucida* on fracture healing in female Wistar rats.

**Methods:**

A drill hole injury was created by inserting a drill bit inthe diaphysis of the femur. The aqueous extract of the whole plant of *Peperomia pellucida* was administered orally at the doses of 100, 200 and 400 mg/kg to adult female Wistar rats. The vehicle (distilled water) was given to the control. Besides these rats, one group of rats without fracture received the extract (400 mg/kg). After 14 days of treatment, the rats were sacrificed under anesthesia and the effects of the extract were evaluated on body weight, the relative weights of organs (femurs, uteri and ovaries) and on hematology. Bone (calcium, phosphorus, alkaline phosphatase) and serum biochemical parameters (calcium, phosphorus, alkaline phosphatase) were also evaluated. Radiological and histological tests were carried out on the femurs. The mineral content of the plant extract was also investigated.

**Results:**

The extract induced an increase in body weight at high dose and in WBCs count at low doses. Aqueous extract from *Peperomia pellucida* increased bone calcium at lowest dose but maintained this parameter at normal range at high dose in fractured rat. Alkaline phophatase and phosphorus concentrations reduced significantly (*p* < 0.01) at the dose of 400 mg/kg as compared to fractured rats. Moreover, radiological tests revealed a dose dependent formation of callus at the level of the fracture gap, confirmed by the formation of a highly dense and compact fibrocartilagenous callus. The mineral content of the plant extract revealed the presence of calcium, phosphorus, magnesium, sodium and potassium.

**Conclusion:**

The aqueous extract of *P. pellucida* accelerates bone healing due partly to the mineral content of the extract. These results confirm its traditional use in the treatment of bone fractures.

## Background

A bone fracture is a break in the continuity of the bone [1]. Bone fractures can either be of traumatic origin (motor accidents or falls) or of pathological origin (mainly osteoporosis) in most cases, as a result of bone fragility [2].

A Bone as an organ has the ability to regenerate naturally when a fracture occurs [3]. Generally in modern medicine, treatment of bone fractures involve the use of a cast or plaster to immobilise the fractured limb, followed by the administration of analgesics (pain killers) and anti-inflammatory drugs to patients. The broken bone is then allowed to regenerate naturally on its own [3], since there is no orally active pharmacological agent available for rapid fracture repair [4]. Even though fractures heal naturally, it would be of great interest to discover alternatives to accelerate a bone healing process, thereby reducing the healing period, reducing the cost of treatment and helping patients to quickly regain their good health.

*Peperomia pellucida* (L.)HBK, belongs to the family Piperaceae. It is a herbaceous plant found mainly in America, Africa and Asia. The species develops during rainy periods and thrives in damp, humid soils and under the shade of trees [5].

Traditional uses include treatment of abdominal pain, abscesses, acne, boils, colic, fatigue, gout and rheumatic joint pain [6]. Literature data confirms the hypoglycemic, anti-inflammatory and analgesic effects of *P. pellucida* [7] and equally its antidiabetic [8] properties. Several animal models are used to show bone regeneration among which is drill-hole injury model [4, 9, 10]. We recently demonstrated that, the ethanolic extract of *P. pellucida* promotes fracture healing in rat by anabolic effects on osteoblasts using drill-hole injury model [10]. In addition, this plant contains calcium [11] and metabolites as flavonoids [12] which are known respectively to contribute in matrix deposition during osteogenesis [13] and promote bone formation [12, 14]. The aqueous extract is commonly used in Cameroonian traditional medicine for fracture management. Therefore, we investigated the effects of the aqueous whole plant extract of *P. pellucida* on fracture healing in female rats.

## Methods

### Plant material

A Fresh plant of *Peperomia pellucida* (L.) was harvested in Limbe (Cameroon) in May 2014 precisely in damp areas. The plant was authenticated at the National Herbarium of Cameroon in comparison with the specimen voucher N° 19,555/SRFCam. The whole fresh plant was cleaned, cut into pieces and dried under a shade at room temperature. The decoction was carried out by boiling 100 g of the powder in 1.5 L of tap water for 10 min. The mixture obtained was filtered, frozen at −20 °C and lyophilised at the Institute of Medical Research and Study of Medicinal Plants (MPMI) to yield 28.79%. (*w*/w) which was kept at room temperature until use.

### Evaluation of the mineral composition of *P. pellucida*

Evaluation of the presence of some selected minerals namely: calcium, magnesium, potassium, sodium and phosphorus in the aqueous plant extract was done at the International Institute for Tropical Agriculture (IITA) Yaounde (Cameroon). The mineral constituents of the whole plant of *Peperomia pellucida* aqueous extract were determined using the atomic absorption spectrophotometer as previously described by Benton and Vernon [15]. Briefly, Ca, Mg, K, Na were extracted by dry ashing of 500 mg of the plant extract in a muffle furnace at 500 °C. It was then diluted using 5 ml acid mix of HNO_3_/H_2_O_2_ (1:1) and analyzed with the atomic absorption spectrophotometer. Phosphorus was extracted as above and analyzed using the method previously described by Murphy and Riley [16].

## Animal

Animal studies were conducted in accordance with the approval of the Cameroon National Ethical Committee (Ref n°.Fw-IRb00001954). Three-month-old female Wistar rats weighing between 150 and 200 g were used for the study. They were maintained at room temperature (22 ± 2 °C) on 12 h light-dark natural cycle. Thirty rats were used for this experiment and divided into six groups of five rats in each group. Normal and fractured control (vehicle) groups received distilled water (10 mL/kg); extract treated groups received the plant at three empirically determined doses of 100, 200 and 400 mg/kg by oral route while one normal group was treated with the extract at the dose of 400 mg/kg.

## Drill hole injury in the femur

A drill-hole injury was created in animals as previously described [4, 9, 10]. Briefly, the front skin of the mid femur in rats was incised straight and longitudinally at 1 cm in length under anesthesia. After splitting the muscle, periosteum was stripped to expose the femoral bone surface. A drill-hole injury was created using a drill machine by inserting a drill bit in the anterior portion of the diaphysis of one femur. The administration of distilled water or the plant extract at different doses began the next day after induction of the fracture. Once awake, animals limped (due to the pain in the fractured limb). After wound healing (3–4 days) animals were physically in good health. Plant extract at the doses of 100, 200 and 400 mg/kg were administered orally for 14 consecutive days (8.am-9.am; in the animal house). Twelve hours before autopsy, all animals were put under dry fasting. The rats were sacrificed under anaesthesia using ketamine (30 mg/kg) and valium (10 mg/kg) via intraperitoneal route. The injured femur (right) was collected and fixed in 10% formalin for histological and radiological analysis. Left (uninjured femur) collected was refrigerated at −20 °C for bone homogenates using 0.1 M PBS. The bones of all animals were subsequently subjected to biochemical assays and histopathological examination.

## Bone tissue homogenates

The homogenates were done using 0.2 g of bone (femur) for 3 mL of phosphate buffer saline (PBS) [17]. The proximal portion of each femur was cut, weighed (0.2 g) and ground on a grinding stone, which had beforehand been covered entirely with a hard, transparent, plastic paper. A volume of 3 mL of PBS was added to the paste and the resulting mixture was put in a test tube. The tissue homogenates were centrifuged at 3000 rpm at 4 °C for 30 min. The supernatant obtained was aliquoted and stored in a refrigerator at −20 °C until analyses bone parameters (bone calcium, phosphorus and alkaline phosphatase).

## Effects on haematological parameters

Before sacrifice, rat blood was collected in ethylenediaminetetraacetic acid (EDTA) tubes by pricking the tip of the tail. An automated blood cell count was carried out at the School of Medical Technicians’ laboratory (Yaounde).

## Effects on biochemical parameters

The arteriovenous blood of rats was collected in dry tubes after decapitation under anesthesia. The samples were allowed to rest for about 20 min, and then centrifuged at 3000 rpm for 15 min at 4 °C. The serum collected was stored at −20 °C for biochemical analysis (calcium, phosphorus, alkaline phosphatase,) using commercial kits.

## Bone radiography: effects of *Peperomia pellucida* aqueous extract on bone callus formation

The bones previously conserved in 10% formalin were cleaned from their surrounding soft tissues. The bones were placed on an X-ray film on a table and exposed to X-rays emitted by a generator or x-ray tube (Allengers) for 5 milliseconds. The characteristics of the X-generator were as follow: maximum voltage 125 Kv, main voltage 55Kv, filtration 0.9 Al/75 (permanent) at a distance of 1 m. The X-rays penetrated the bone and images were received by a detector (AGFA, CR 30-Xm) on the examination table. The images were then developed; the diameter of the hole if present in each bone was measured using software (AGFA, CR-30X). Then, the bones were returned immediately inside 10% formalin for histological analysis.

## Bone histology

The bones previously conserved in 10% formalin were demineralized in 10% HCL solution for 5 days. Isolated femur samples containing the drill hole were embedded in paraffin and a thickness of 10 μm sections were made using a microtome (Reichert-Jung 2030). Histological analysis at the site of the fracture was assessed by Picrosirius staining method. Photographs of the sections were taken using a digital camera for microscope (DCM 35:350 K Pixels, USB 2.0) aided with appropriate filters.

## Statistical analysis

Data are expressed as the mean ± standard error mean. Statistical significance was determined by one-way ANOVA followed by Dunnet as the post-test using the Graphpad Prism software version 5.03. Differences were considered significant at *p* < 0.05.

## Results

### Mineral composition of *P. pellucida*

Analyses of the mineral content of the dry extract of this plant revealed the presence of some minerals including potassium (K), phosphorus (P), magnesium (Mg), calcium (Ca) and sodium (Na) (Table 1). K was found to be the most abundant mineral element in this plant with a mass of 16.640 μg/g, followed by P with 1.405 μg/g. Ca was found to weigh 0.454 μg/g while Na was the least with a weight of 0.181 μg/g.

**Table 1:** Mineral composition of the aqueous extract of *P. pellucida*

Minerals	Mass (μg/g)
Potassium (K)	16.640
Phosphorus (P)	1.405
Magnesium (Mg)	0.801
Calcium (Ca)	0.454
Sodium (Na)	0.181

### Effects of the aqueous extract of *P. pellucida* on body weight

**Fig. 1:**
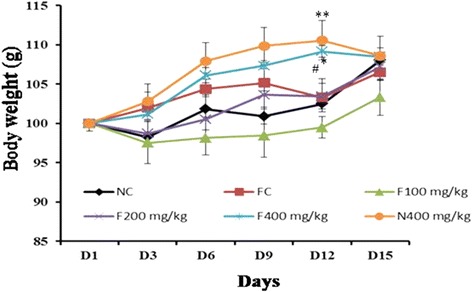
Effects of the aqueous extract of *P. pellucida* on body weight of rats. The values represent the mean ± SEM, *n* = 5. * *p* < 0.05,** *p* < 0.01,: significant difference compared to NC. #*p* < 0.05, significant difference compared to FC. NC = Normal control rats, FC = Fracture control rats. F100 mg/kg, F200 mg/kg and F400 mg/kg = Fractured rats treated with the aqueous extract of *P. pellucida* at doses of 100, 200 and 400 mg/kg respectively, N400 mg/kg = Normal rats treated with *P. pellucida* at the dose of 400 mg/kg

Figure 1 represents the effects of the aqueous extract of *P. pellucida* on body weight of rats. Bone fracture induction in animals led to a non-significant increase in body weight with respect to the normal control, during the 14 days of experiment. Administration of the plant extract to the fractured and unfractured rats at the dose of 400 mg/kg induced a significant increase (*p* < 0.01, *p* < 0.05 respectively) in body weight on the 12th day of treatment as compared to their respective controls.

### Effects of the aqueous extract of *P. pellucida* on haematological parameters

The effects of *P. pellucida* on differential blood counts in rats are summarized in Table 2. Administration of the plant extract at a dose of 100 mg/kg led to a significant increase (*p* < 0.01 and *p* < 0.05) in the number of white blood cells (WBCs) by 91.92% and 52.64% as compared to the normal control and fractured control respectively. The plant extract at the dose of 200 mg/kg led to a significant increase (*p* < 0.01) by 60% in the amounts of WBCs with respect to the fractured control. At the dose of 400 mg/kg for the fractured rats, there was a significant decrease (*p* < 0.05) in the granulocyte count by 23.62% and a significant increase (*p* < 0.001) in the value of MPV by 290.6% when compared to the normal control. The administration of the plant extract to the non-fractured rats led to a significant increase (*p* < 0.001, *p* < 0.01, *p* < 0.01 and *p* < 0.001 respectively) in the values of MID, MCH, MCHC and MPV respectively as compared to the normal control. There was a significant decrease (*p* < 0.05) in the value of GRA count by 45.15%.

**Table 2:** Effects of *P. pellucida aqueous extract* on hematological parameters

Parameters	NC	FC	F100 mg/kg	F200 mg/kg	F400 mg/kg	N400 mg/kg
WBCs(10^3^/uL)	11.27 ± 1.1	14.17 ± 0.9	21.63 ± 2.0**#	22.74 ± 6.7***###	8.270 ± 1.6	12.30 ± 1.6
LYM(10^3^/uL)	4.713 ± 0.6	5.633 ± 0.8	4.577 ± 0.3	4.550 ± 0.4	4.103 ± 1.1	6.120 ± 0.1
MID(10^3^/uL)	1.303 ± 0.03	1.243 ± 0.5	1.230 ± 0.01	1.263 ± 0.03	1.853 ± 0.7	4.367 ± 0.1***###
GRA(10^3^/uL)	7.767 ± 0.9	5.250 ± 0.8	5.257 ± 0.6	5.000 ± 0.9	4.017 ± 1.2*	4.267 ± 0.2*
RBCs(10^3^/uL)	4.557 ± 0.3	5.317 ± 0.8	4.437 ± 0.4	4.467 ± 0.4	4.643 ± 0.2	5.210 ± 0.2
HCT(%)	40.40 ± 0.7	40.43 ± 1.9	40.17 ± 1.8	40.70 ± 0.3	44.77 ± 2.9	40.30 ± 0.8
MCV(fl)	88.67 ± 3.3	86.07 ± 0.6	87.47 ± 2.3	84.80 ± 0.4	96.00 ± 5.7	100.7 ± 9.9
RDW(%)	18.27 ± 2.0	21.80 ± 2.0	16.27 ± 1.6	23.30 ± 3.0	16.20 ± 2.0	15.37 ± 2.7
MCH(Pg)	37.07 ± 4.3	37.77 ± 3.0	35.43 ± 3.0	29.57 ± 3.2	40.90 ± 2.0	55.90 ± 3.2**##
MCHC(g/dl)	40.17 ± 3.5	37.30 ± 3.4	35.10 ± 5.5	39.37 ± 6.9	47.40 ± 1.7	66.40 ± 6.0**##
HGB(g/dl)	12.87 ± 0.5	12.70 ± 0.5	12.32 ± 0.8	12.40 ± 0.23	13.50 ± 0.6	12.98 ± 1.7
PLT(10^3^/uL)	457.7 ± 81.6	439.7 ± 101.5	315.3 ± 82.1	386.3 ± 94.7	283.0 ± 10.3	327.0 ± 18.0
MPV(fl)	14.47 ± 2.1	12.20 ± 1.6	18.10 ± 1.3	11.93 ± 0.9	47.60 ± 4.9 ***###	48.27 ± 4.7***###

Values represent the mean ± SEM, *n* = 5, *p < 0.05**, *p* < 0.01, *** *p* < 0.001: significant difference compared to NC. #*p* < 0.05,## *p* < 0.01, ### *p* < 0.001: significant difference compared to FC. *NC* Normal control rats, *FC* Fracture control rats. F100 mg/kg, F200 mg/kg and F400 mg/kg = Fractured rats treated with the aqueous extract of *P. pellucida* at the doses of 100, 200 and 400 mg/kg respectively, N400 mg/kg = Normal rats treated with *P. pellucida* at the dose of 400 mg/kg. *WBCs* White blood cells, *LYM* Lymphocytes, *MID* Minimum inhibition dilution, *GRA* Granulocytes, *RBCs* Red blood cells, *HCT* Haematocrits, *MCV* Mean corpuscular volume, *RDW* Red cell distribution width, *HGB* Haemoglobin, *MCH* Mean corpuscular haemoglobin, *MCHC* Mean cell haemoglobin concentration, *PLT* Platelets, *MPV* Mean platelet volume

### Effects of the aqueous extract of *P. pellucida* on serum and bone calcium concentrations

**Fig. 2:**
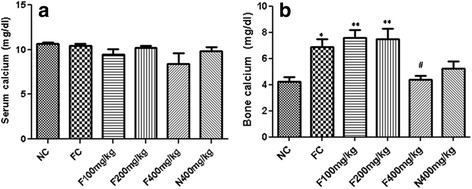
Effects of the aqueous extract of *P. pellucida* on serum (**a**) and bone (**b**) calcium concentrations in rats. Each bar represents the mean ± SEM (*n* = 5). * *p* < 0.05, ** *p* < 0.01 significant difference compared to NC. # *p* < 0.05 significant difference compared to FC. NC = Normal control rats, FC = Fracture control rats. F100 mg/kg, F200 mg/kg and F400 mg/kg = Fractured rats treated with the aqueous extract of *P. pellucida* at doses of 100, 200 and 400 mg/kg respectively, N400 mg/kg = Normal rats treated with *P. pellucida* at the dose of 400 mg/kg

The effects of the aqueous extract of *P. pellucida* on serum and bone calcium concentrations are summarized on Fig. 2. Fracture induction after two weeks did not provoke a significant change in serum calcium concentration in rats. Also, the administration of the plant extract at all doses during this experimental period, showed a non-significant decrease in the concentrations of serum calcium as compared to their control (Fig. 2a). However in bone (Fig. 2b), fracture induction led to a significant increase (*p* < 0.05) by 63.39% of bone calcium compared to the normal control. The administration of aqueous extract of *P. pellucida* at the doses of 100 and 200 mg/kg to fractured animals did not show a significant modification of bone calcium compared to fractured rats. However, the plant extract at the same doses induced a significant increase (*p* < 0.01) in bone calcium concentrations, by 9.55% and 8.22% respectively, as compared to the normal control. Treatment of fractured rats with extract at the dose of 400 mg/kg rather led to a significant decrease (*p* < 0.05), by 36.48% of this parameter as compared to the fractured control. It is important to mention that the plant extract at the dose of 400 mg/kg did not significantly modify serum and bone calcium in non fractured rats as compared to the normal control.

### Effects of the aqueous extract of *P. pellucida* on serum and bone phosphorus concentrations

**Fig. 3:**
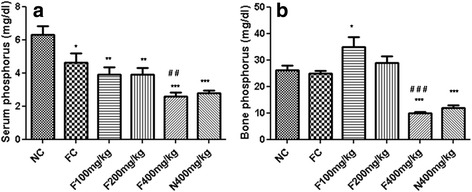
Effects of the aqueous extract of *P. pellucida* on serum (**a**) and bone (**b**) phosphorus concentrations in rats. Each bar represents the mean ± SEM (*n* = 5). * *p* < 0.05, ** *p* < 0.01, *** *p* < 0.001 significant difference compared to NC. #*p* < 0.05,## *p* < 0.01, ### *p* < 0.001 significant difference compared to FC. NC = Normal control rats. FC = Fracture control rats, F100 mg/kg, F200 mg/kg and F400 mg/kg = Fractured rats treated with the aqueous extract of *P. pellucida* at doses of 100, 200 and 400 mg/kg respectively, N400 mg/kg = Normal rats treated with *P. pellucida* at the dose of 400 mg/kg

Figure 3 represents the effects of the aqueous extract of *P. pellucida* on serum and bone phosphorus concentration in rats. Fractured control rats receiving distilled water showed a significant decrease (*p* < 0.05) by 26.78%, in the serum phosphorus concentration as compared to the normal control (Fig. 3a). The administration of the plant extract at the doses of 100, 200 and 400 mg/kg in fractured rats, induced a significant decrease by 15.44%, 15.16% and 44.42% respectively as compared to the normal control. The plant extract induced a significant decrease (*p* < 0.001) in this parameter as compared to the normal control. Treatment of fractured animal with the plant extract at lower doses (100 and 200 mg/kg) did not significantly alter the serum phosphorus concentration as compared to the fractured control. However, at the dose of 400 mg/kg there was a significant decrease in phosphorus concentration (*p* < 0.01) by 59.30%. In bone, the plant extract at the dose of 100 mg/kg led to a marked increase (*p* < 0.05) by 33.52% in phosphorus concentration as compared to the normal control (Fig. 3b). When compared to the fracture control, this increase was by 40.41%. At the dose of 400 mg/kg the decrease was by 60.19% in comparison with the fracture control. When compared with the normal control, unfractured rats treated with the plant extract also showed a significant decrease (*p* < 0.001) by 54.67% in the bone phosphorus concentrations.

### Effects of the aqueous extract of *P. pellucida* on serum and bone alkaline phosphatase (AP) concentrations

**Fig. 4:**
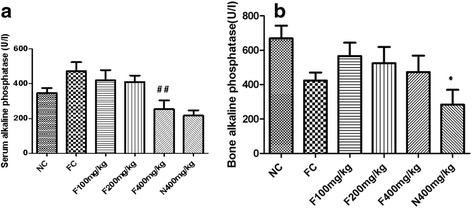
Effects of the aqueous extract of *P. pellucida* on serum (**a**) and bone (**b**) concentrations of AP in rats. Each bar represents the mean ± SEM (*n* = 5). * *p* < 0.05, significant difference compared to NC. ## *p* < 0.01, significant difference compared to FC. NC = Normal control rats, FC = Fracture control rats, F100 mg/kg, F200 mg/kg and F400 mg/kg = Fractured rats treated with the aqueous extract of *P. pellucida* at doses of 100, 200 and 400 mg/kg respectively, N400 mg/kg = Normal rats treated with *P. pellucida* at the dose of 400 mg/kg

The effects of the aqueous extract of *P. pellucida* on serum and bone AP concentrations are represented in Fig. 4. Animals presenting bone injury showed a non-significant increase in serum AP activity as compared to the normal control (Fig. 4a). Treatment of fractured rats with the plant extract at the dose of 400 mg/kg, led to a significant decrease (*p* < 0.01) by 42.23% in AP activity as compared to the fracture control. In bone, treatment of non-fractured rats with the plant extract at the dose of 400 mg/kg induced a significant decrease (*p* < 0.05) by 57.61% with respect to the normal control (Fig. 4b).

### Effects of the aqueous extract of *P. pellucida* on bone callus formation in rats: radiography and histology

**Fig. 5:**
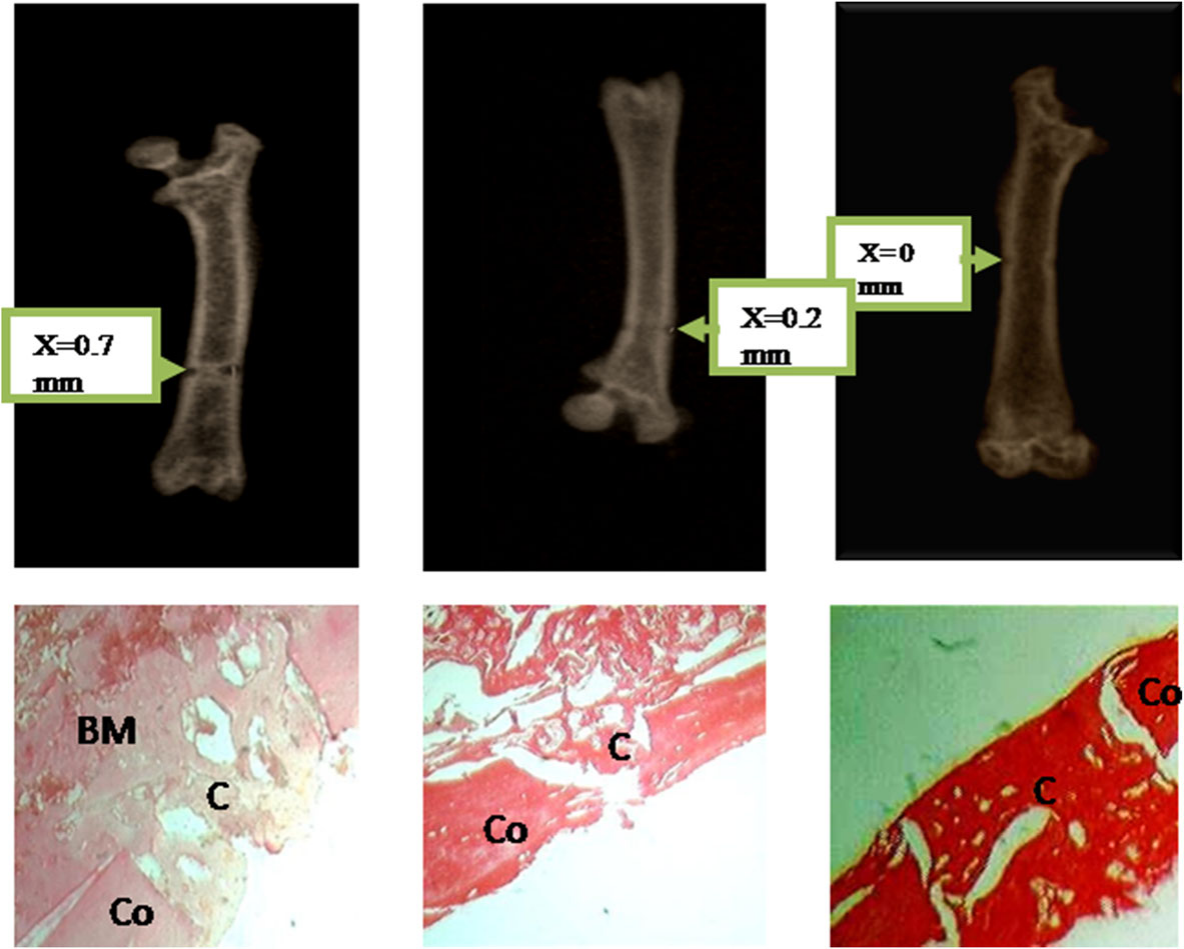
Effects of the aqueous extract of *P. pellucida* on bone callus formation (Picrosirius staining, × 25). BM: Bone marrow, Co: Cortical bone and C: Callus. F200 mg/kg and F400 mg/kg = Fractured rats treated with the aqueous extract of *P. pellucida* at doses of 200 and 400 mg/kg respectively

Effects of *P. pellucida* on bone callus formation are summarized in Fig. 5. The results of bone X-rays revealed that the treatment of fractured rats with the plant extract at the doses of 100, 200 and 400 mg/kg induced a dose dependent decrease in the drill hole diameter to 0.5, 0.2 and 0 mm respectively. This corresponds to 28.75, 71.43 and 100% decrease respectively in comparison to the fractured control. There was complete closure of the hole in the rats receiving the plant extract at 400 mg/kg. These results are confirmed in histology where fractured control showed callus formation in the drill hole with a loose arrangement. Administration of the plant extract led to a dose dependent deposition of the callus with a slightly compact callus at the dose of 200 mg/kg while at 400 mg/kg, the callus was found to be more compact and dense as compared to the fracture control.

## Discussion

*Peperomia pellucida* is a herb used in Cameroonian traditional medicine to treat fracture. However, no systematic study is available to confirm the efficacy of the aqueous extract of this plant in accelerating fracture healing. Till date, there is no orally available agent/compound used to treat fractures. In the present study, we firstly evaluated the mineral content of the aqueous whole plant extract of this plant. Minerals and most especially calcium and phosphorus (hydroxyapatite) build up the inorganic content of bones [18]. In this study, results of the analyses of the mineral content of the aqueous extract of *P. pellucida* reveal the presence of some minerals like potassium, phosphorus, magnesium, calcium and sodium. These results are in accordance with those of Ooi *et al.* [11] who found out that *P. pellucida* harvested in Malaysia, contained these similar minerals. The presence of these minerals in *P. pellucida* therefore makes it an appropriate dietary source of bone minerals making us to suggest that the administration of this plant extract to both unfractured and fractured rats could help to build up strong bones by providing them with essential minerals important in mineralization of osteoid, during bone formation [13].

The results of body weight of animals during the experimental period reveal that, the administration of the plant extract at the dose of 400 mg/kg induces an increase in body weight of fractured and unfractured rats. These results suggest that, *P. pellucida* aqueous extract could enhance appetite at higher doses. The red marrow in the femur is the principal site of hematopoiesis in the body [19]. Thus, an injury to this organ may affect the haematopoietic process. Bone haematology revealed that the RBCs and platelets counts remained within the normal range after the 14 days period of treatment. This indicates that the plant extract did not have any relevant effect on these blood cells production, and suggesting maintenance of the bone marrow integrity. However, the administration of the plant extract at the doses of 100 and 200 mg/kg led to a significant increase in WBCs levels suggesting an inflammatory response [19] of the extract. To confirm this suggestion, further studies need to be performed. On the other hand, it is known that thrombocytes promote bone regeneration and their activation and function can be measured as mean platelet volume (MPV) [20]. The plant extract at high dose tripled the MPV in fractured and unfractured rats. This result suggests the ability of the plant extract to promote bone regeneration by accelerating migration and proliferation of osteogenic cell in fractured rat. However in unfractured rat the increase in MPV confirms the capability of the plant extract to act on thrombocyte activation and function which could be beneficial when fracture occurs. On the contrary some authors have shown that, the increase in MPV could be related to problems in the union of fracture in human [21]. *P. pellucida* plant has been shown to possess osteogenic compounds like flavonoids and phytoestrogens [2], which have the ability to stimulate the recruitment of osteoblasts at the injury site [9] and to increase the amount and activities of osteoblasts [22] respectively. The increased activity of osteoblasts leads to the rapid deposition of osteoid or soft callus (which fills the fracture gap) which is later on mineralised, thereby adding weight to bone. Calcium level is an important marker of bone healing. The creation of the fracture resulted in a reduced serum calcium levels and a high bone calcium level. These results could indicate a mobilisation of calcium ions from serum to bone. It has been shown that, calcitonin is a hormone secreted as a negative feedback mechanism in response to an increase in plasma calcium concentration [23], an increase which could be due to the high calcium content of the plant extract. The results of mineral composition of the extract confirm this assertion. In bones, the results show that the plant extract at the doses of 100 and 200 mg/kg favoured calcium mineralisation. This result is in consonance with a previous report [10] which showed that *P. pellucida* ethanolic extract dose-dependently increased mineral deposition. This could be attributed not only to the presence of calcium in the extract but equally to the presence of phytoestrogens and flavonoids in the extract [2] which stimulate osteoblastic activities. These active osteoblasts in turn secrete high amounts of alkaline phosphatase and osteocalcin which are important in bone mineralisation [19] hence, calcium deposition. Conversely to the doses of 100 and 200 mg/kg, the plant extract at the dose of 400 mg/kg in both fractured and normal rats, induced a non-significant decrease in levels of calcium when compared to the normal control. This may suggest bone healing during which, osteoblasts activities and secretions return to their normal levels [24]. This view correlates with the reduction in AP activities and complete closure of fracture gap (X-rays examination) at this dose as observed in these groups.

Phosphorus is an essential bone-forming element because it is required for the appropriate mineralisation of the skeleton [25]. Evaluation of phosphorus levels in animals revealed that, bone phosphorus concentrations are higher than serum levels. This could be due to an accumulation of phosphorus in bones which is a reservoir for this mineral [18]. In bones, the extract induced an increase in the deposition of this mineral at the doses of 100 and 200 mg/kg. This could possibly be as a result of the high phosphorus content of our extract. In fact, during a bone healing process, calcium and phosphorus crystals derived from nutritional sources are deposited in bones in the form of hydroxyapatite [13] in the phase of bone mineralisation. This is in accordance with the results of calcium levels which have also increased in these groups. The increase mineralisation could equally be attributed to the presence of copper [11] in the extract which enhances bone formation and skeletal mineralization [25]. In the groups receiving the plant extract at the dose of 400 mg/kg, there was a decrease in the bone mineral content to baseline values which could be due to the reduction in the activity of osteoblasts after fracture healing [24], leading to a decrease in mineralisation an hence in phosphorus deposition. This goes in line with the results of alkaline phosphatase activity and calcium content which equally reduced after administration of the plant extract at the dose of 400 mg/kg during 14 days.

The detection of specific serum biomarkers of bone formation, such as alkaline phosphatase can be clinically useful in evaluating the progress of a healing process. Alkaline phosphatase is secreted by osteoblasts during the maturation phase of bone formation [13]. Fracture induction provokes a decrease in bone alkaline phosphatase as observed in this study. When the plant extract was administered, there was a non-significant increase in bone alkaline phosphatase activity. This result suggests that the aqueous extract of *P. pellucida* could accelerate the maturation phase of osteoblasts. In addition, the presence of zinc in this extract [11] could enhance osteoblastic activity and alkaline phosphatase activity [25]. It is important to note that, at the dose of 400 mg/kg in fractured rats, the increase in alkaline phosphatase activity was lower than that observed at lower doses. This could indicate bone healing during which the osteoblastic activities and secretions decrease to reach the normal value. Some studies confirm a decrease in bone specific alkaline phosphatase to baseline values during natural normal fracture healing [24, 26]. X-rays examination helps to assess a bone healing process [27]. The results obtained after x-rays examination at the site of the fracture reveal that, 14 days after the fracture, the callus formed is already observable. In the fracture control group, the diameter of the hole has slightly reduced. This indicates that the natural process of bone healing has occurred [3]. The administration of the extract accelerates a fracture healing process which is dose dependent. There is a decrease in the diameters of the fracture gaps in the treated groups with complete closure in the rats treated with the highest dose (400 mg/kg). These results suggest the powerful effect of the plant extract to fill up the gap. This was made evident with the histology realised at the fracture sites. In fact after 14 days of fracture induction, animals presented a loose bone structure which confirms once again a natural bone regeneration. When administered, the plant extract led to more packed and dense cells suggesting an acceleration of a bone repair process. Previous works done by Ngueguim *et al.* [10] showed that ethanol extract of *P. pellucida* significantly increases the expression of BMP-2 genes. Thus, the rapid healing process observed, could be due to an increase in the expression of BMPs which are potent inducers of osteogenesis leading to fracture healing [13].

## Conclusion

The results revealed that the plant extract contains varied nutrient minerals (calcium, phosphorus) which are important for bone health. It was observed that, administration of the extract to fractured animals lead to an increase in body weight. The extract equally induced a significant increase in WBCs count at the doses of 100 and 200 mg/kg and MPV at the dose of 400 mg/kg; a significant increase in the amounts of calcium, phosphorus and AP. From the results it was observed a dose dependent effect of *P. pellucida* on callus formation. These results suggest that, the aqueous whole plant extract of *P. pellucida* possesses osteoblastic effects responsible for enhancing bone repair process.

## Abbreviations

ALP: Alkaline phosphatase
EDTA: Ethylenediaminetetraacetic acid
GRA: Granulocytes
HCT: Haematocrits
HGB: Haemoglobin
LYM: Lymphocytes
MCH: Mean corpuscular haemoglobin
MCHC: Mean cell haemoglobin concentration
MCV: Mean corpuscular volume
MID: Minimum inhibition dilution
MPV: Mean plasmatic volume
PBS: Phosphate buffer saline
PLT: Platelets
RBCs: Red blood cells
RDW: Red cell distribution width
WBCs: White blood cells
